# Robust and accurate quantification of biomarkers of immune cells in lung cancer micro-environment using deep convolutional neural networks

**DOI:** 10.7717/peerj.6335

**Published:** 2019-04-10

**Authors:** Lilija Aprupe, Geert Litjens, Titus J. Brinker, Jeroen van der Laak, Niels Grabe

**Affiliations:** 1 Hamamatsu Tissue Imaging and Analysis (TIGA) Center, BioQuant, Heidelberg University, Heidelberg, Germany; 2 Department of Medical Oncology, National Center for Tumor Diseases (NCT), University Hospital Heidelberg, Heidelberg, Germany; 3 Department of Pathology, Radboud University Medical Center, Nijmegen, The Netherlands; 4 Steinbeis Center for Medical Systems Biology (STCMSB), Heidelberg, Germany; 5 Department of Dermatology and National Center for Tumor Diseases (NCT), University Hospital Heidelberg, Heidelberg, Germany

**Keywords:** Lung cancer, Immune cells, Deep learning, Cancer micro-environment, Biomarker quantification

## Abstract

Recent years have seen a growing awareness of the role the immune system plays in successful cancer treatment, especially in novel therapies like immunotherapy. The characterization of the immunological composition of tumors and their micro-environment is thus becoming a necessity. In this paper we introduce a deep learning-based immune cell detection and quantification method, which is based on supervised learning, i.e., the input data for training comprises labeled images. Our approach objectively deals with staining variation and staining artifacts in immunohistochemically stained lung cancer tissue and is as precise as humans. This is evidenced by the low cell count difference to humans of 0.033 cells on average. This method, which is based on convolutional neural networks, has the potential to provide a new quantitative basis for research on immunotherapy.

## Introduction

Tumors contain not only malignant cells but also diverse non-malignant cells, such as those from the immune, vascular and lymphatic system, in addition to fibroblasts, pericytes, extracellular matrix and adipocites. These cells can even comprise more than 50% of the mass of the primary tumors and their metastases (Balkwill, Capasso & Hagemann, 2012). To date it is not well-studied how the non-malignant cells in the tumor micro-environment regulate tumor progression and its response to treatment (Pietras & Östman, 2010; Herbst et al., 2014; Hoefflin et al., 2016; Michel et al., 2008).

Especially since the introduction of new, highly effective, immunotherapy strategies, which try to get immune cells to attack the tumor, the interest in accurate quantification of immune cells in the tumor and its micro-environment has substantially increased (Vesely et al., 2011; Fridman et al., 2012; Chen & Chefd’Hotel, 2014; Carstens et al., 2017). Accurate quantification could potentially allow for new biomarkers which can help predict therapy success and monitor therapy progression (Guo et al., 2015; Varn et al., 2017). But the benefit of such automated quantification tools is not limited to just immunotherapy, the immune cell density and their localization in the proximity of cancer might help to predict the presence and development of the metastases and overall survival of cancer patients (Halama et al., 2011; Mlecnik et al., 2016; Van Den Eynde et al., 2017). Moreover, previous work has shown that T-cell density (detected by staining of the CD3 and CD8 cell markers) is an essential parameter for predicting a success of a chemotherapy (Halama et al., 2011).

Manual immune cell counting on a microscopic tissue section is tedious, time-consuming and subjective, and thus unsuitable for analysis of large number of images within the scope of clinical studies. The utilization of computer-assisted scoring of immune cell infiltrates, especially in multi-institutional studies and clinical trials, has clear advantages due to its reproducibility and automation potential (Zu et al., 2005; Sander et al., 2014).

Since every stained cell could look different due to biological variability, it is often very challenging to judge, which immune cells are sufficiently stained to count them as a “positive stained cell”. This occurs even if the stain is sufficiently specific and automated staining systems are used. Therefore, a robust computational solution is necessary for the analysis of variably stained images to obtain robust decision boundary, which holds for every image and is not reconsidered in others, for example, images obtained from other laboratories.

Nowadays, multiple companies provide software tools for histological image analysis. However, usually tedious and time consuming software parameter tuning on different images and staining conditions are necessary. Several computational nuclei and cell segmentation algorithms exist, however they are usually bound to the imaging technique, tissue and staining type (Veta et al., 2013). Multiple processing steps are necessary to achieve a good segmentation and these approaches usually avoid regions with severe infiltration of lymphocytes, necrosis and focusing artifacts (Friedrich et al., 2016). Often the anthracotic pigment on lung histological slides provides an additional layer of complexity, since deciphering between round spots of an anthracotic pigment and a cell is not a trivial task. Due to these issues, manual cell counting methods are often still more precise than these conventional image analysis methods (Veta et al., 2013).

In this work we utilize a deep learning method, multilayer convolutional neural networks. The recent advancement in learning algorithms and the availability of the graphics processing units (GPUs) has lead to the dominance of the deep learning method in the image analysis and computer vision fields (Krizhevsky, Sutskever & Hinton, 2012; LeCun, Bengio & Hinton, 2015; Wang et al., 2016; Fakhry, Peng & Ji, 2016). These networks learn features directly from pixels values in an image. The neural networks mathematically models a consecutive chain of neurons (nodes) and their synapses (weights) (McCulloch & Pitts, 1943; Rosenblatt, 1962). Convolutional neural networks are a type of neural networks, where the weights are shared between the neurons, so that the overall operation of neurons is similar to convolution.

Recently deep learning approaches were introduced to different subjects in digital pathology: mitotic cell detection (Cireçan et al., 2013), nuclei detection (Sirinukunwattana et al., 2016), growth pattern classification (Anthimopoulos et al., 2016), patient stratification (Bychkov et al., 2018) and immune cell detection (Chen & Chefd’Hotel, 2014). These inspiring initial approaches, to our knowledge, focused mostly on classifying tiles within whole-slide images. Recently, several others have applied convolutional neural networks to whole-slides directly, for example for detection and segmentation of breast cancer metastases in lymph nodes (Litjens et al., 2016; Turkki et al., 2016; Wang et al., 2016). A recent work of Turkki et al. (2016) detected immune cell-rich tissue regions based solely on the haematoxylin and eosin stained cell morphology. Moreover, the study of Chen & Chefd’Hotel (2014) performed stained immune cell detection using deep learning. However, we believe we add to scientific community with our work by showing that our method is applicable to whole slide setting, that we are able to use tissue image data without extensive preprocessing and by applying fast radial symmetry for the positive stained cell counting to achieve a human-like performance in positive cell counting. To our knowledge deep learning technique has not yet been applied specifically to immune cell counting in histological whole slide images.

In this article we present a robust and quantitative immune cell detection system, which could be used for describing immune system involvement in the cancer micro-environment. Automatic stained T-cell analysis, which is presented in this paper, is based on the deep learning on the manually labeled input images. The network was trained and applied on several immune cell biomarkers without training the network on each biomarker (CD3, CD8 and CD20) separately.

## Methods

### Dataset

To gain an insight into the state of the tumor and its environment a fragment of a tumor tissue is collected from the patient by a resection. Afterwards the tumor tissue fragments were immunohistochemically stained, digitalized and analyzed with computerized image analysis techniques and manually.

We used lung adenocarcinoma tissue stained for markers CD3, CD8 and CD20, which stain all T-cells, cytotoxic T cells and B-cells respectively. Acquired data in total comprised 39 tissue slides. The tissue slides were provided by the Tissue Bank of the National Center for Tumor Diseases (Heidelberg, Germany). Staining of the tissue samples was performed according to standard staining protocol on a Bond Autostainer (Leica) with anti-CD3 (SP7, monoclonal rabbit; Abcam), anti-CD8 (SP16, monoclonal rabbit, Zytomed Systems) and anti-CD20 (L26, monoclonal mouse; Leica). The tissue samples were counterstained with hematoxylin. Detection was performed with Bond Polymer Refine Detection kit with DAB. The tissue glass slides were digitalized with a Nanozoomer 2.0 (Hamamatsu) slide scanning system at a resolution 0.228 µm/px at 40×  magnification.

**Figure 1:**
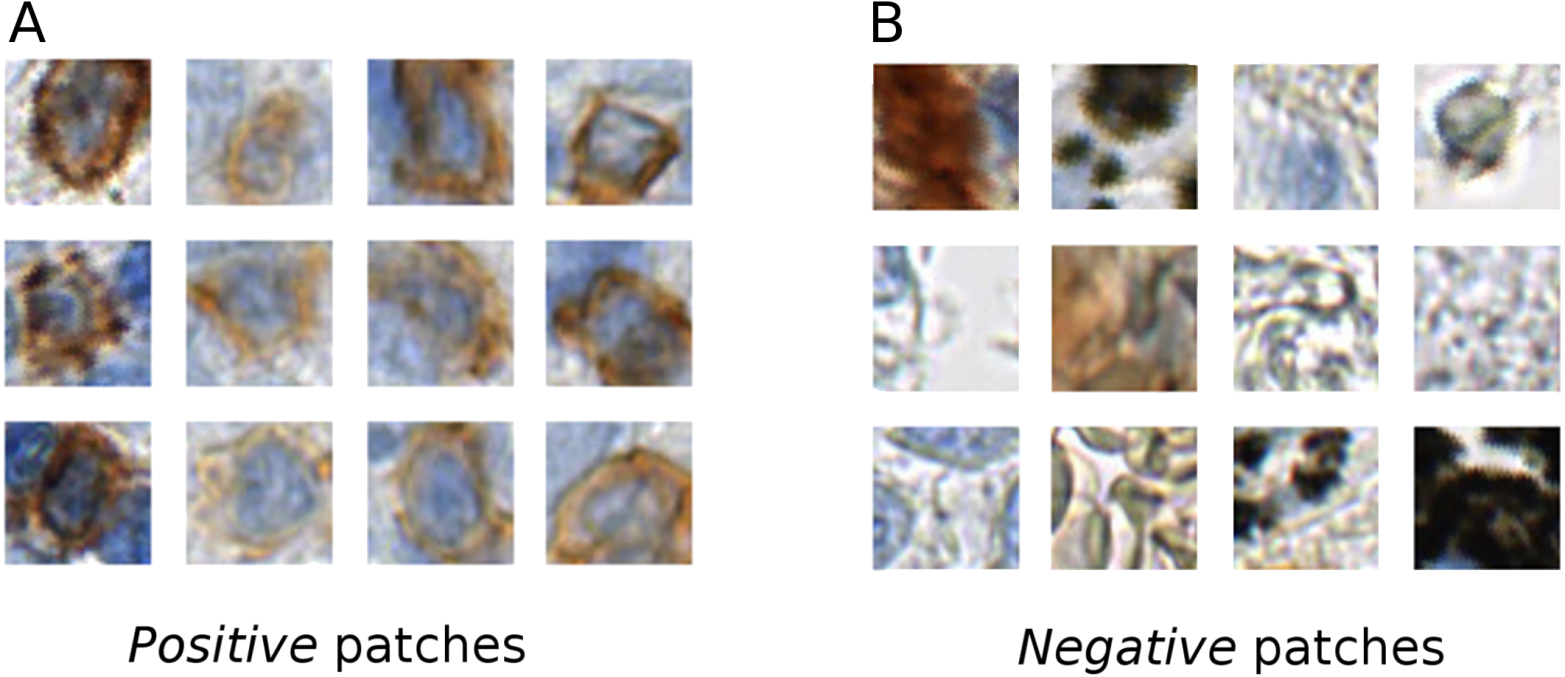
Example of the positive (A) and negative (B) patches of a training image set. It is evident that the *positive* cells have varying morphology and also show varying staining intensity, from dark to light brown, whereas the *negative* class is highly variable, involving erythrocytes, anthracotic pigment, hematoxylin, diffuse stain traces and others.

The tissue areas, which contained stained immune cells, were manually annotated by a biologist (L.A.) using the NDP.view2 software (Hamamatsu). From the positive annotations we extracted 1,500 ×  1,500 px regions, which contained stained immune cells. In these regions we manually annotated all the centers of the positive stained cells, from which RGB patches of (46 ×  46 px) were extracted centered at the annotated point. These patches are used as a training data for the positive class (Fig. 1). We also manually annotated tissue regions without positively stained cells. From these regions we directly randomly sampled 46 × 46 pixel RGB patches. These patches were used as examples for the negative class (Fig. 1).

Our training data was collected considering various factors, such as, stain color intensity among *positive* cells and cell morphology (see Fig. 1). In our training set’s *negative* class we included not only anthracotic pigment, but also various unspecifically stained cells, morphological tissue irregularities and stain “leaks” (Fig. 1). These patches served as a basis for two class-based supervised training of the neuronal network.

We split the dataset in two: 27 slides for training and 12 slides for testing. We used 9 slides of each stain (CD3, CD8 and CD20) for training and 4 slides for testing the training progression. Negative areas were obtained from these slides where no positively stained cells were present. The patches were augmented by mirroring them horizontally and vertically and rotating by 40 degrees. In total each class contained about 800 thousand patches. For training we took 1,224,000 patches from the 27 training slides (as an input for the convolutional network model) and from these 12 testing slides we took 408,000 patches as a static validation set to monitor training progression.

### Network training

**Figure 2:**
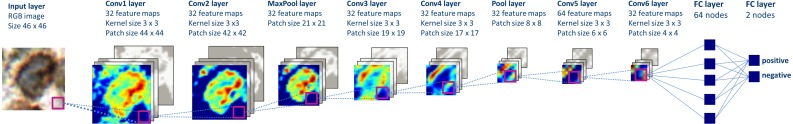
The structure of the deep convolutional neural network, which was applied to image classification. The patches are propagated through the network, in which the consecutive convolutional and pooling operations are applied, thus the number of nodes is reduced downstream. Two final layers perform input classification. Heatmaps depict activations of the filters of respective layer of the network.

We trained multiple deep convolutional neural network models using open-source libraries Theano 0.8 and Lasagne 0.2 (Bergstra et al., 2010; Bastien et al., 2012; Dieleman et al., 2015). Best performing neural network was comprised of six convolutional, two pooling layers and two fully connected layers (Fig. 2). The network was trained using stochastic gradient descent (gradient descent optimization using a few stochastically chosen training examples) with a learning rate of 0.01. For accelerating gradient descent we used Nesterov momentum of 0.9. The network training was stopped after one pass over all training patches as subsequent passes did not improve validation set results.

The performance of the network was tested with respect to classification accuracy of the network on the patch level and the network performance in cell counting tasks compared to humans.

Confusion matrix, false positive and false negative rates, sensitivity and specificity were calculated using 13,817 randomly selected validation patches.

## Results

We trained the deep convolutional network on the training set, which was built of patches belonging to two classes: positive class (T-cells) and negative class (other cells and artifacts) (Fig. 1). The training was performed with the network structure (Fig. 2) and parameters mentioned in the Methods section.

**Figure 3:**
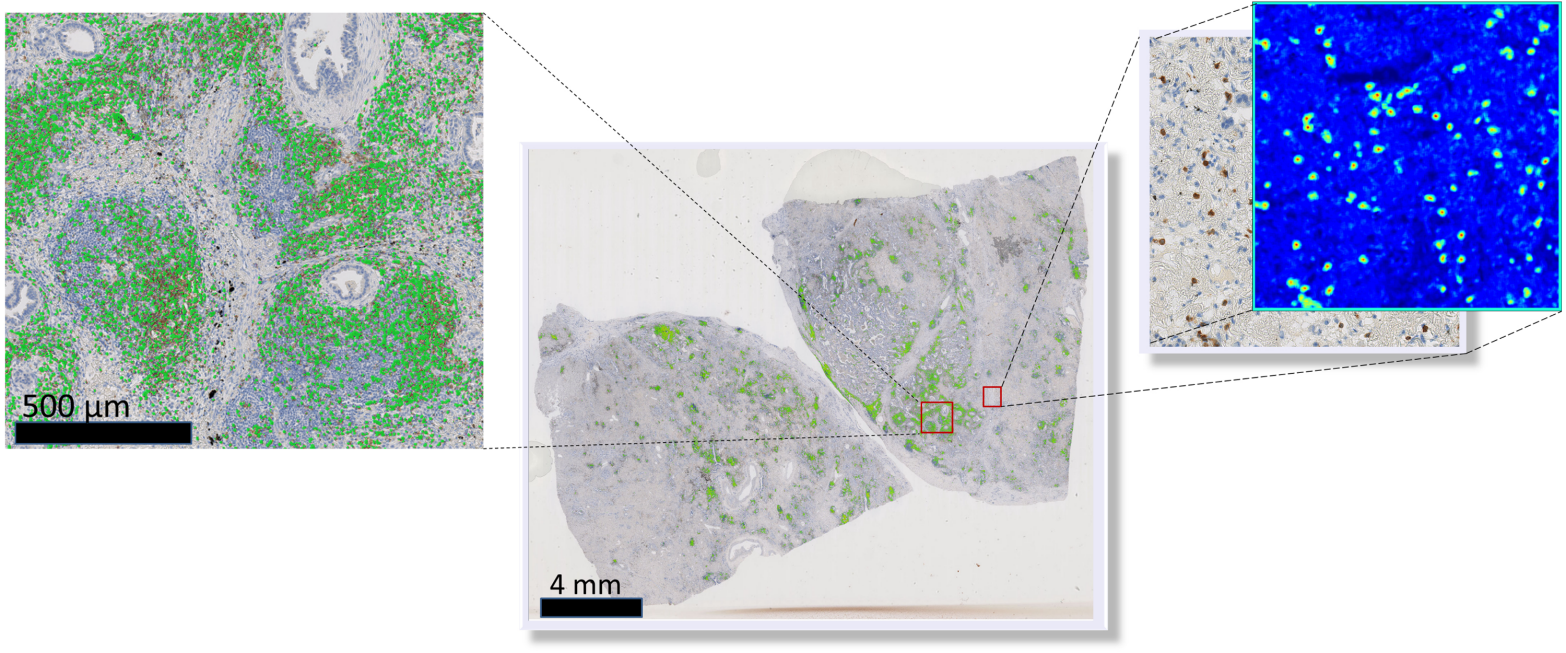
Schematic representation of the immune cell detection map (green dots) on a whole slide image, which is generated by overlaying original digital slide image with a posterior likelihood map (heatmap with blue background).

To visually access network classification accuracy on whole slide level, we generated likelihood maps on several whole slide images. The neural network model was applied on a pixel-by-pixel basis on a whole digital slide, yielding a posterior likelihood of a every pixel of being a positive cell (Fig. 3), thus generating an immune cell localization likelihood map.

**Figure 4:**
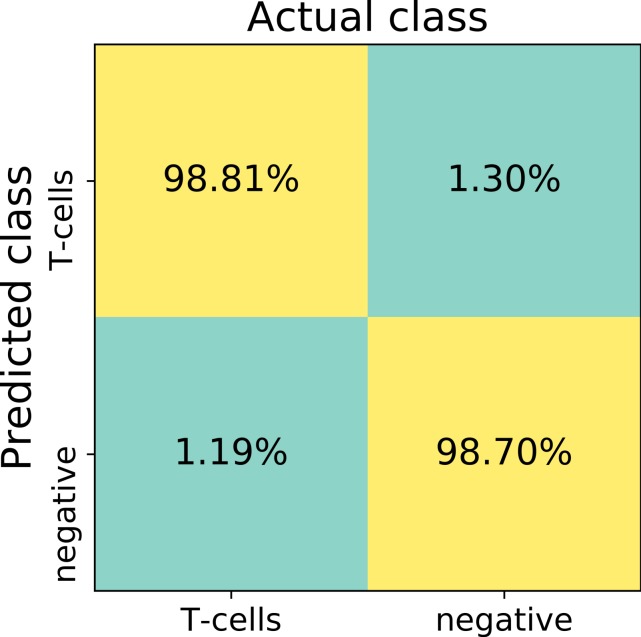
The confusion matrix summarizes true positive (upper left), true negative (lower right), false positive (upper right) and false negative (lower left) cases of detection on a patch level. Numbers in squares represent number of cases in percent normalized to total number of patches in a labeled (actual) class. In total, 13817 validation patches contributed for calculation of the detection statistics.

The first trained network recognized false positive objects on the whole slide images, therefore we expanded our training set with exceptionally challenging cases of negative class. For this we extracted additional negative patches from the false positive detected areas. We used both former negative patches and the newly extracted false positive cases to augment our training set of the deep neuronal network. After subsequent training, the model obtained a validation set accuracy of 98.6% on the augmented patch level. The sensitivity in the discrimination of T-cells on the patch level was 98.8%, whereas specificity 98.7%. False negative detection rate is 1.19% and 1.30% for false positive detection (Fig. 4). For examples of false positive and false negative cases, see Fig. S1.

We also quantitatively accessed our model’s output with respect to human performance. For this we created an independent set of validation images. We randomly selected 64 large square images from the 12 testing/validation whole slide images. The area of single randomly selected image is 0.2 mm^2^, corresponding to 2,000 × 2,000 px. In total, testing images summed up to the tissue area of 13.6 mm^2^.

We created the likelihood map for every validation image by applying the trained neural network on the validation image on a pixel-by-pixel basis. On the likelihood map we detected positive nuclei centers using a fast radial symmetry transform (FRST) (Loy & Zelinsky, 2003; Veta et al., 2013). FRST is a fast gradient-based feature detection method, which identifies points of high radial symmetry. We set parameters for FRST as follows: α (radial strictness) to 0.5, β (a gradient threshold) to 0.25, minimum and maximum radius of nuclei was set to 0 and 4 µm respectively, the threshold for the likelihood map was set to 0.3. Subsequent thresholding of the FRST resulted in a set of connected components that were regarded as individual positive nuclei. FRST helped to remove false positive detected objects from the final result and obtain the coordinates of the detected immune cells.

**Figure 5:**
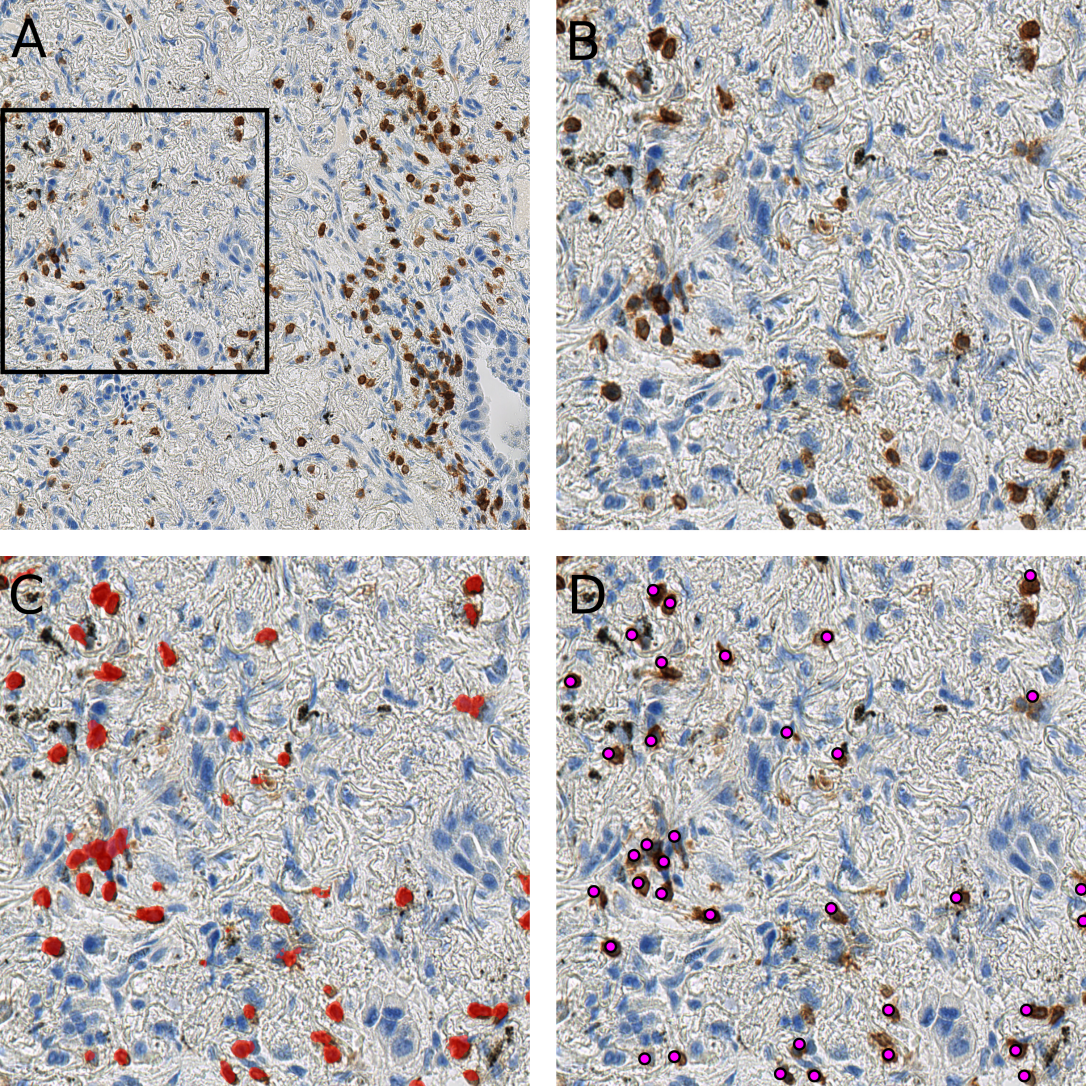
Presentation of the deep learning method’s performance on the validation slides. In (A) a testing image randomly selected from a whole-slide image stained for CD3^+^ cells (dimensions: 2,000 × 2,000 px; Size 0.2 mm^2^). Validation was performed on 64 such images. A frame is maginified in (B) on the right; (C) represents the likelihood map created with the trained network, which is overlayed with the image in (B). Cells, which are recognized by the model, are marked red. Anthracotic pigment (black), a common artifact, is not detected; (D) shows detected cells (magenta dots) after application of the fast radial symmetry transform algorithm on the likelihood map.

As shown in the Fig. 5, the randomly selected testing set also includes images where anthracotic pigment and staining artifacts are present, and also stained immune cells are of varying staining intensity, size and shape. Fig. S2 contains images regions from validation set, where different staining artifacts and how our method deals with them are shown.

However, Fig. 5C clearly shows, through the likelihood maps, that the network manages to ignore this anthracotic pigment and to precisely detect immunohistologically stained immune cells in the lung adenocarcinoma tissue samples. Figure 5D presents the resulting overlay of the histological image and detected objects after the fast radial symmetry method.

**Figure 6:**
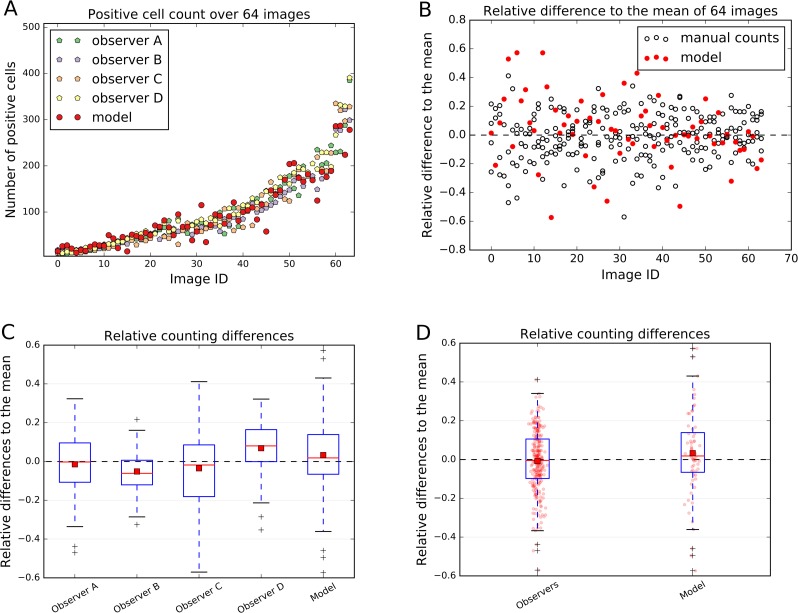
Comparison of the model performance to four independent human observers. (A) shows number of positive cells detected by model (red) or counted by observers in the validation images. (B) shows the relative differences of the model to the mean of the observers’ manual counts. The data in (B) is summarized in boxplots in (C) and (D). In (A) and (B) the *x*-axis represents images, which are sorted in order from smallest to largest number of mean counted cells. The dashed line in (B), (C) and (D) represents the mean counted cells over all images and all observers; the red squares in (C) and (D) show the mean. White circles in (B) represent counts of a person on a respective image, whereas the filled red circles represent the measurements of a model. In (D) red dots represent counts of cells in validation images.

The count of the immune cells obtained by the deep neural network was compared to the number of manually counted cells by human observers. The human “gold standard” was obtained by four independent human observers. Four observers marked each CD3, CD8, and CD20 positive stained immune cell in these 64 images. The manual counting results in comparison to the model performance are summarized in Fig. 6.

The relative count differences are obtained by subtracting the counted value from the mean over all observations and the model and normalizing it to the number of mean counted cells per image. The distances of the relative difference to the mean measurement line show how far the measurement is from the consensus measurement.

We show that the variation of manually counted cells among observers is considerable (Figs. 6B and 6C). The overall agreement of the cell counts varies among the observers, some being close to the overall mean, but some (e.g., Observers B and D) are not; e.g., the interquartile range (IQR) of the *Observer B* lies within [−0.120, 0.006] and of *Observer D* within [−0.001, 0.164] (Fig. 6C).

In the Fig. 6C and 6D it is also evident, that the model is performing within the variation of the manual observer. The box-and-whisker plot in the Fig. 6D shows the relative difference to the mean across all 64 testing images for each observer and the model. The IQR_Observers_ = [−0.097, 0.106] is strongly overlapping with the automatically detected cell counts IQR_Model_ = [−0.066, 0.139] (Fig. 6D). The mean of the automatic method is close to the mean over all observers and model’s cell counts (Average_Model_ = 0.033) (Fig. 6C).

We can observe that the relative differences of the model to the mean is higher, if the number of cells in an image is small (Fig. 6B). We also observe higher variability in observer cell counts in cases where stained immune cell clusters are present.

**Figure 7:**
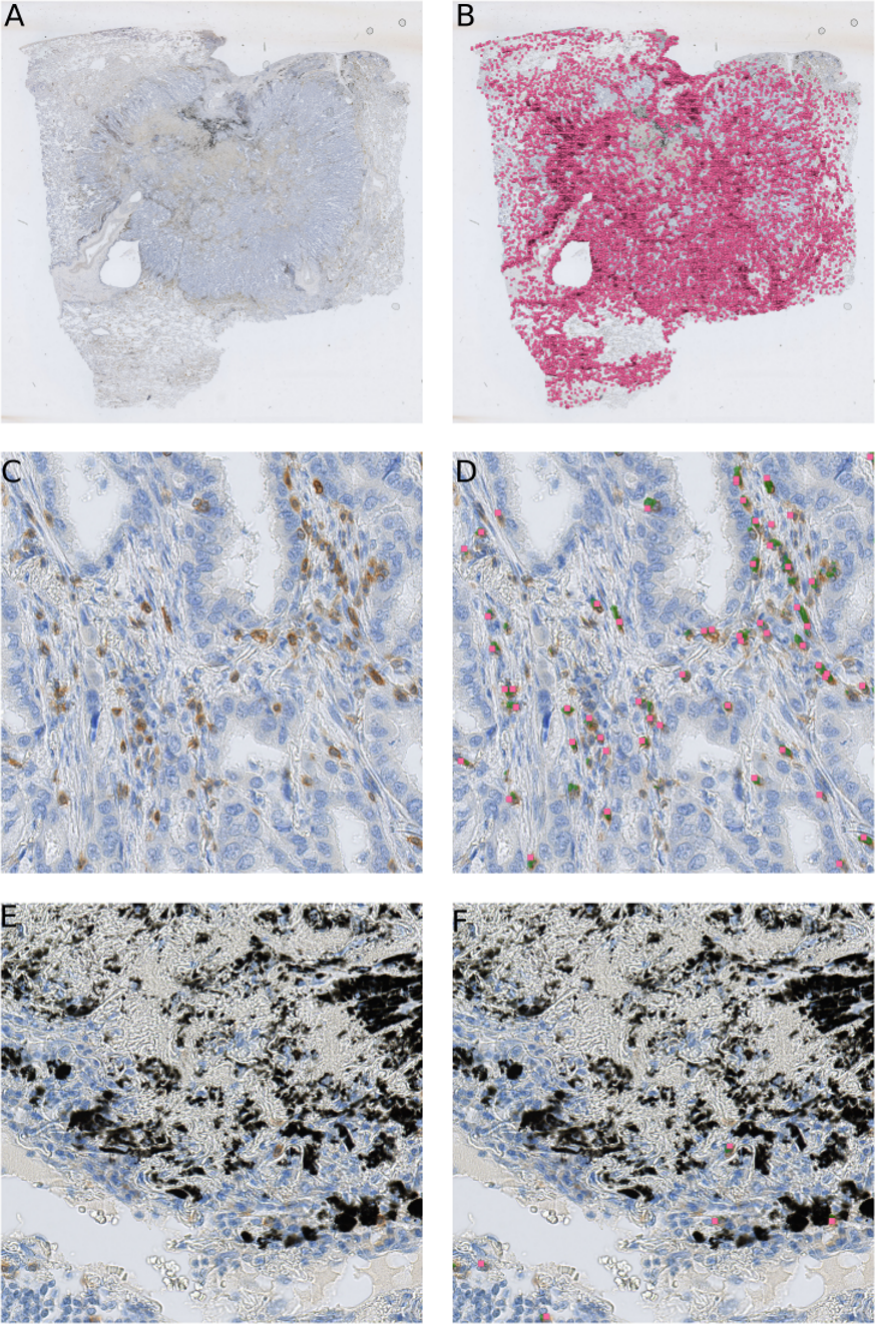
Our cell detection method applied to a whole slide image of a tissue stained with an anti-CD3 antibody. (A) A whole-slide image (WSI) of a lung adenocarcinoma sample. Tissue area is about 301 mm^2^. (B) detected T-cells in (A) are marked with magenta squares. (C) A fragment of a tissue from (A) with stained T-cells (CD3 marker, brown). (D) Overlay of (C) with likelihood map (green) and final detected cells (magenta squares). (E) A fragment of a tissue from (A) with an anthracotic pigment (black). (F) Overlay of (E) with likelihood map (green) and final detected cells (magenta squares).

Our method is not limited to small images only. We are able to obtain similar results on the whole-slide level (Fig. 7), providing reliable comparison between patient tissue samples. Here we examined CD3 biomarker stained immune cells of a lung resection of a patient, which was not included either in the training set or testing set. Our method recognizes vaguely stained cells and identifies anthracotic pigment as an artifact (Fig. 7F).

## Discussion

Here we presented a deep learning-based method for automated and robust detection of immune cell biomarkers in immunohistochemically stained tissue of human lung adenocarcinoma. The presented method enables comparison of multiple tissue sections with respect to absolute or relative counts of infiltrated leukocytes in a whole-slide setting.

The power of the method lies within its robustness, applicability to different tissue samples and cell types from different biomarker stains. This reduces the need to optimize the system for each different type of stain. Moreover, our method performed well with respect to tissue artifacts, such as, anthracotic pigment, cell staining variations, morphological irregularities of the tissue and unspecific staining.

We are aware of inspiring previous studies which use deep learning for tissue profiling in digital pathology. However, our study differs from the other studies, which are mentioned in the introduction, since we used one classifier for different cell types (T-cells and B-cells) and we focused on the detection and counting of single cells. We also performed immunostaining, which was not considered by some of the previous works. Also, the subject of our study, the lung adenocarcinoma tissue, exhibits challenges for classification algorithm, especially because of the presence of the anthracotic pigment. Further strengths of our work is the method’s applicability to the whole slide scale and the fact that no additional training for different stain and image preprocessing is required.

We were able to train a single network for CD3, CD8 and CD20 without re-optimizing for each stain. Furthermore, our network was able to deal with very challenging tissue areas, such as an anthracotic pigment. Namely, the trained deep learning network did not recognize anthracotic pigment particles as a T-cells. Therefore, a single neural network model can be applied to a number of images, which may include anthracotic pigment, scanning and staining artifacts, large stained cell clusters and tissue heterogeneity, without extensive adaption of the parameters for specific images.

Our approach was validated on a randomly selected and independent testing set, which was manually evaluated by four independent observers. The cell counts estimated by our method showed concordance with the cell counts obtained manually, as evidenced by the low difference with the relative mean of 0.033 units distance from the overall mean on average. Thus our method is able to reproduce human-like performance in cell counting.

However, the limitations of this work include the fact that all the data is obtained from one type of scanner also staining was performed in a single laboratory, thus our setting does not represent a multi-center study. Another limitation is the inaccurate network’s performance on the clustered cell populations. Leukocyte detection in highly infiltrated regions is a commonly reported issue in digital pathology (Turkki et al., 2016). We observed a trade-off between specificity in detecting spread cells and sensitivity on clustered cell populations. If we modify FRST in favor of clustered cells, algorithm is sensitive on clustered cells but not specific enough in other sites of the tissue, where cells are not clustered as densely. To overcome this problem, our approach could be combined with methods which specifically deal with clustered cell populations (Halama et al., 2009).

Our automatic method enables quantification of immune cells in different tissue regions, such as, the tumor and invasive margin, which might provide a precious information for oncological treatment decisions. In the future the registration of multiple whole slide images could open a new staining and analyzing paradigm, which could make use of consecutively stained tissue samples, e.g., colocalizing different stains on a cell level, thus finding cells, which are positive for multiple markers in a cancer tissue.

Our method is applicable to other tumor micro-environmental entities, e.g., macrophages, fibroblasts, which can be specifically stained by means of immunological stains. This approach allows to combine multiple models in a “tumor micro-environment map”, where one could overlay a “cancer detection map” with ”a detection map” of cell population expressing various markers.

## Conclusions

The immune cell counting technique presented in this work can be applied for developing a robust and multifaceted description of the cancer micro-environment in research applications as well as in the clinical practice. This approach could help pathologists to quantify immune cells on a whole slide level more precisely and time-effectively and step towards novel stratified immune therapies in personalized oncology.

##  Supplemental Information

10.7717/peerj.6335/supp-1 Supplemental Information 1 The showcase of how well deep learning model performs on unknown examples (patches and validation images) Figure S1 indicates patches which model could not discriminate properly, such as, false positive and false negative cases. Figure S2 shows an overview over variety of artifacts, which are encountered within the stained lung adenocarcinoma tissue slides. These images are extracted from the validation image set. In general, artifacts are not recognized as cells by our method. The red dots indicate detected cells.

10.7717/peerj.6335/supp-2 Supplemental Information 2 Coordinates of the positively stained cells The positions of the positive cells were detected by four independent human observers on at least 64 different validation images. Each csv file stored in the zip archive represents the positive cell count and their position per person and validation image.

## Additional Information and Declarations

### Data Availability

The following information was supplied regarding data availability: Our Supplemental File contains selected images from the validation set and false positive and false negative patches.
