# Supplementary material for: Robust and accurate quantification of biomarkers of immune cells in lung cancer micro-environment using deep convolutional neural networks
Source: PeerJ. 2019 Apr 10;7:e6335. doi: 10.7717/peerj.6335 (PMC6462181; doi:10.7717/peerj.6335)

Supplemental Information

Robust and accurate quantification of biomarkers of immune cells in lung cancer micro-environment using deep convolutional neural networks

Lilija Aprupe, Geert Litjens, Titus J. Brinker, Jeroen van der Laak, and Niels Grabe

Supplemental Figure Legends

Figure S1. Images of false positive and false negative patches.

Images show the cases which model could not discriminate properly on the patch level, such as false positive and false negative cases.

(A) False negative cases
(B) False positive cases

Figure S2. Images of common artifacts in the validation patches.
Here we present an overview over variety of artifacts, which we encounter within the stained lung adenocarcinoma tissue slides. These images are extracted from the validation image set. In general, artifacts are not recognized as cells by our method.

(A) Unspecific staining
(B) Anthracotic pigment
(C) Tissue structural, morphological irregularities
(D) Very light staining
(E) Staining variations
(F) Overstaining
(G) Different cell types

Supplementary Figure 1

(A)

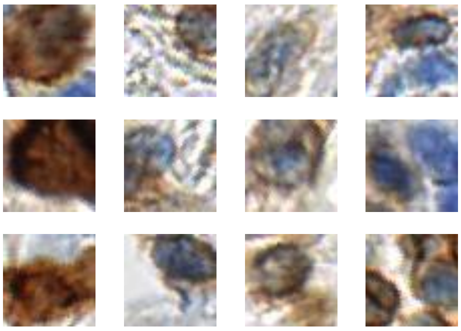

(B)

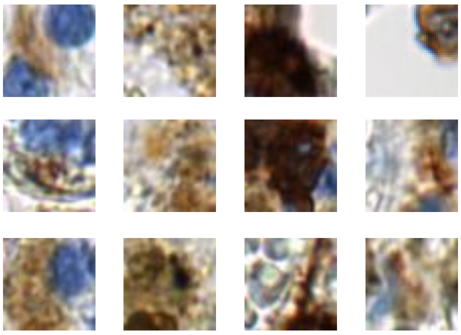

Supplementary Figure 2

(A)

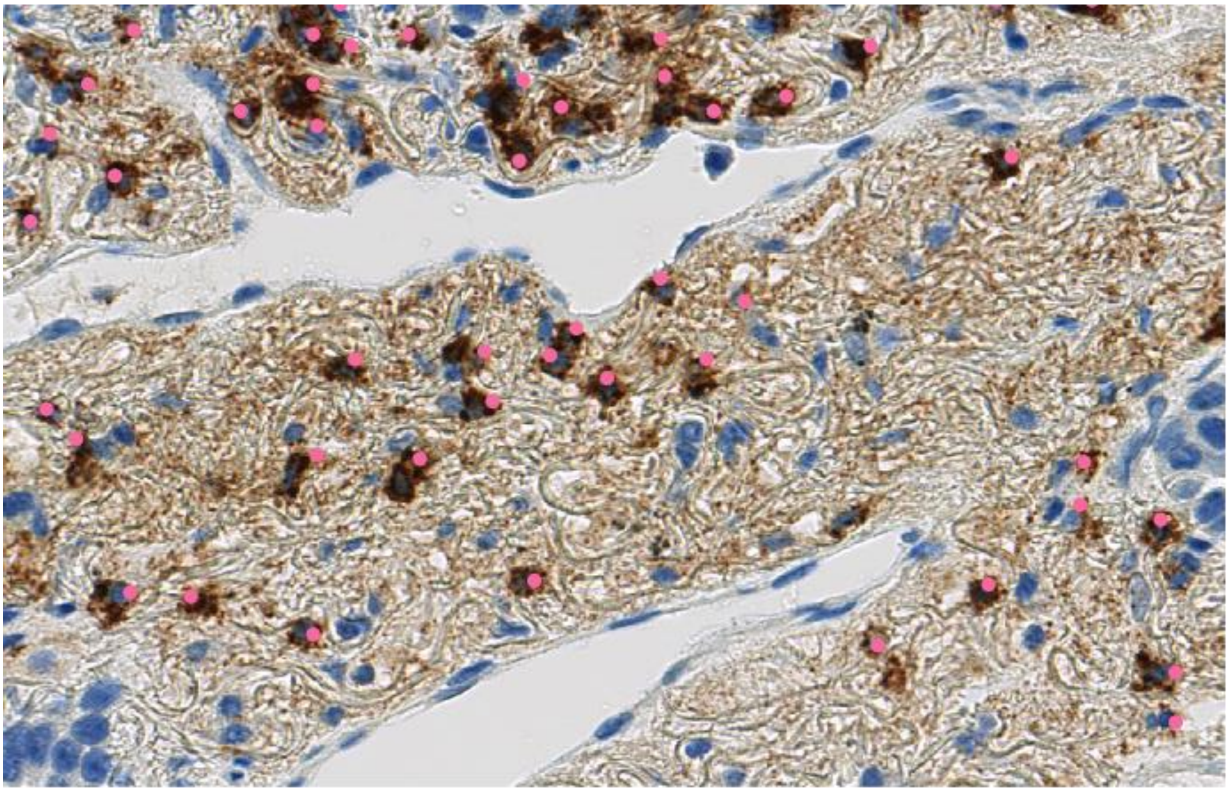

(B)

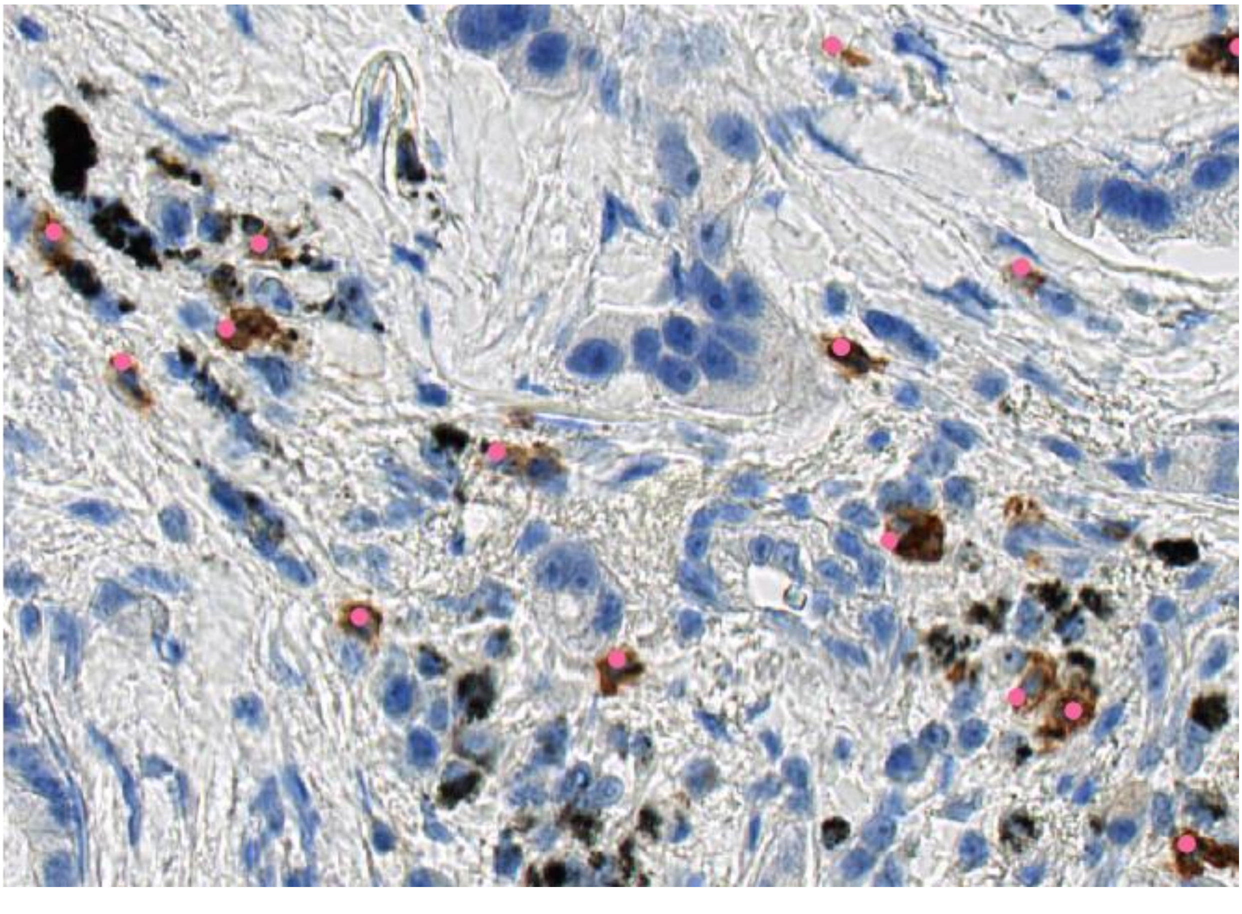

(C)

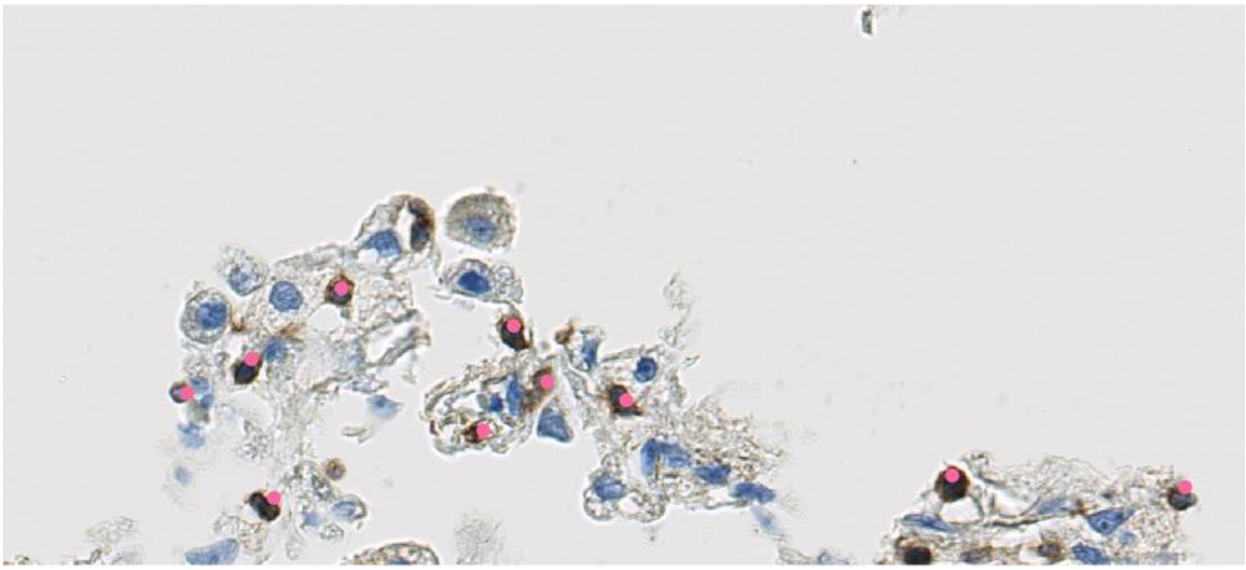

(D)

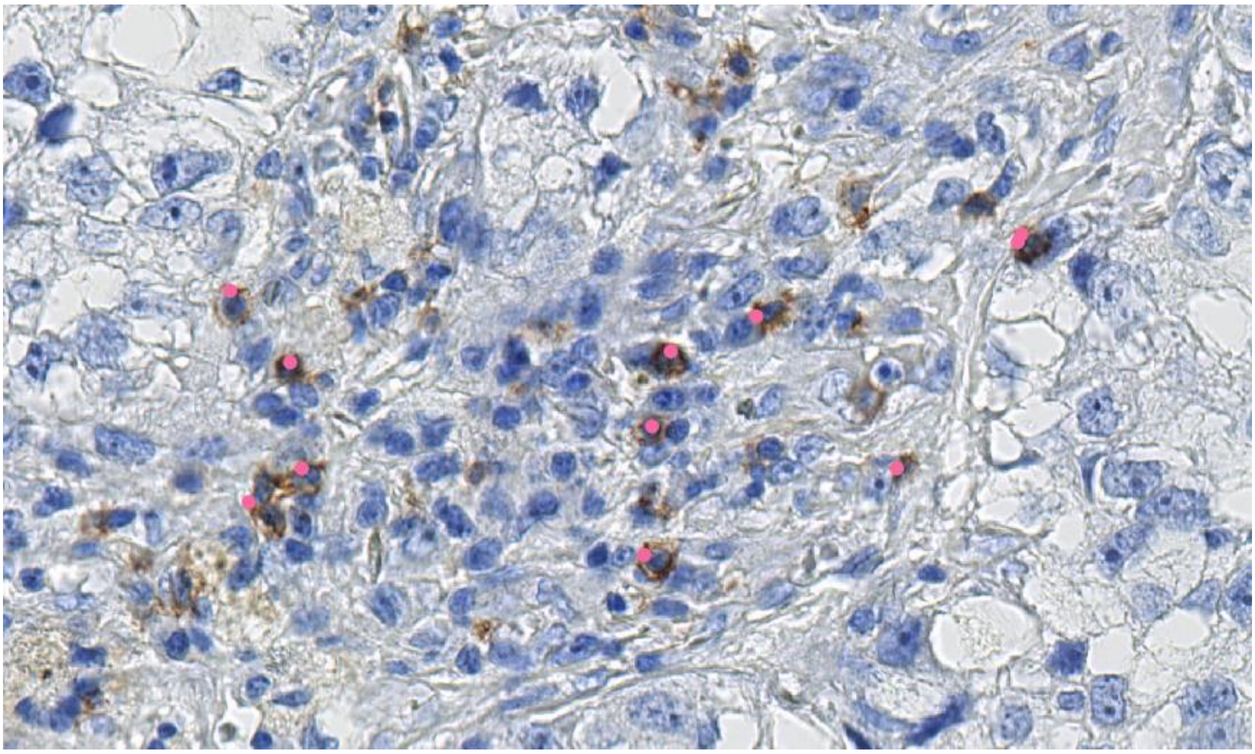

(E)

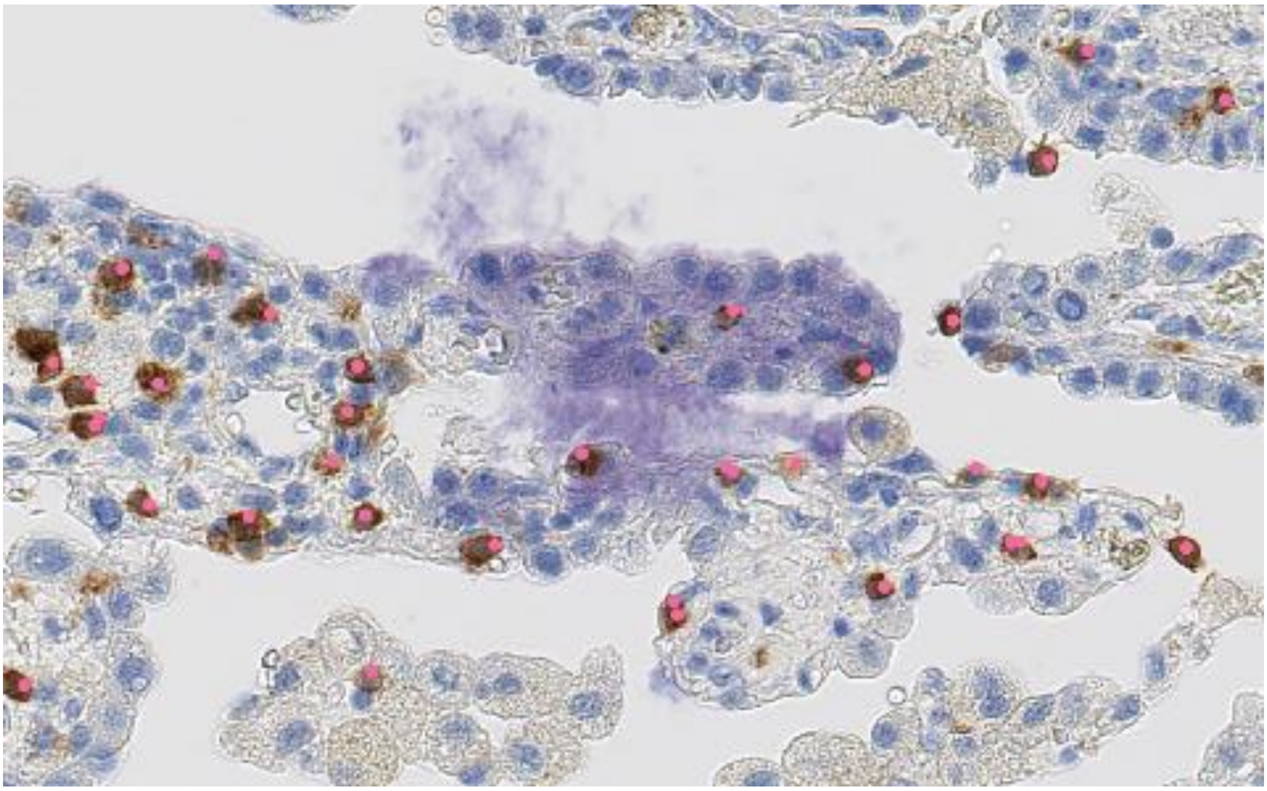

(F)

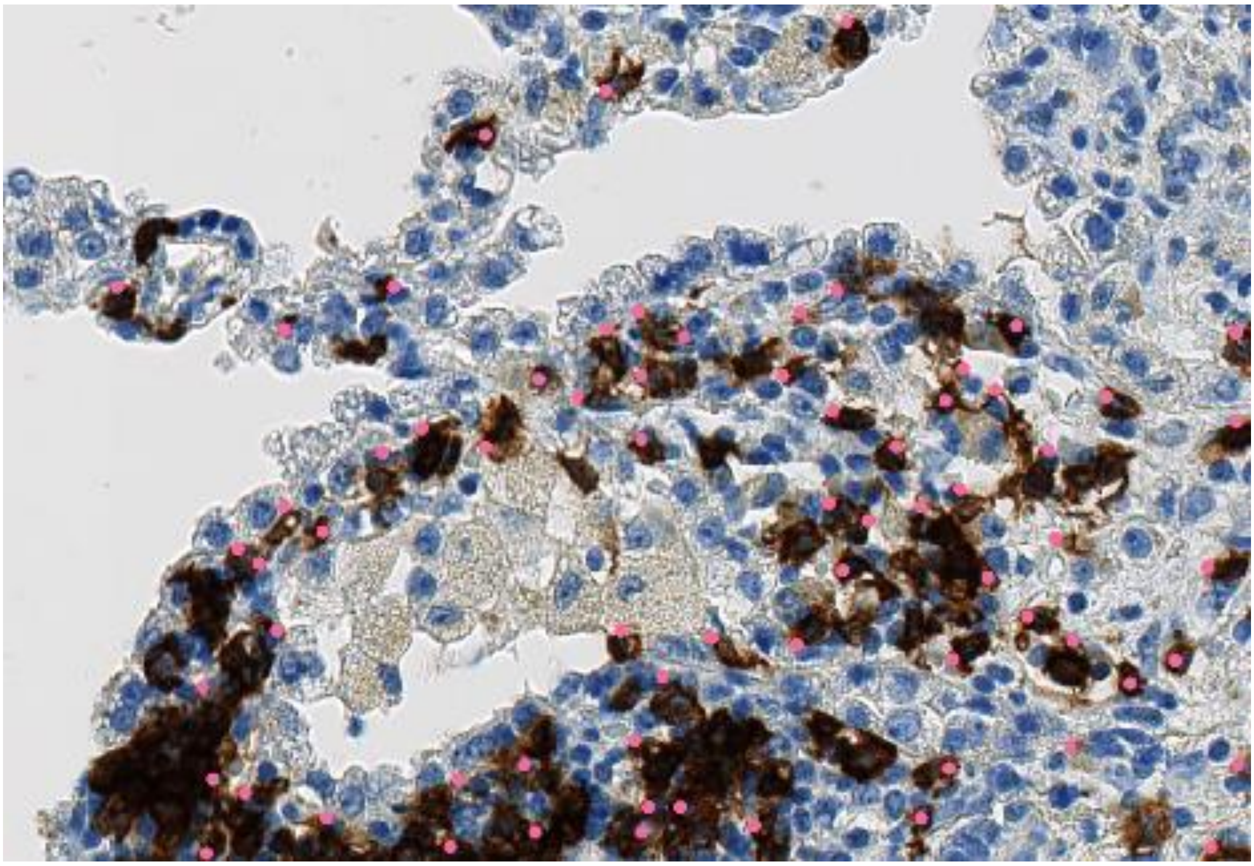

(G)

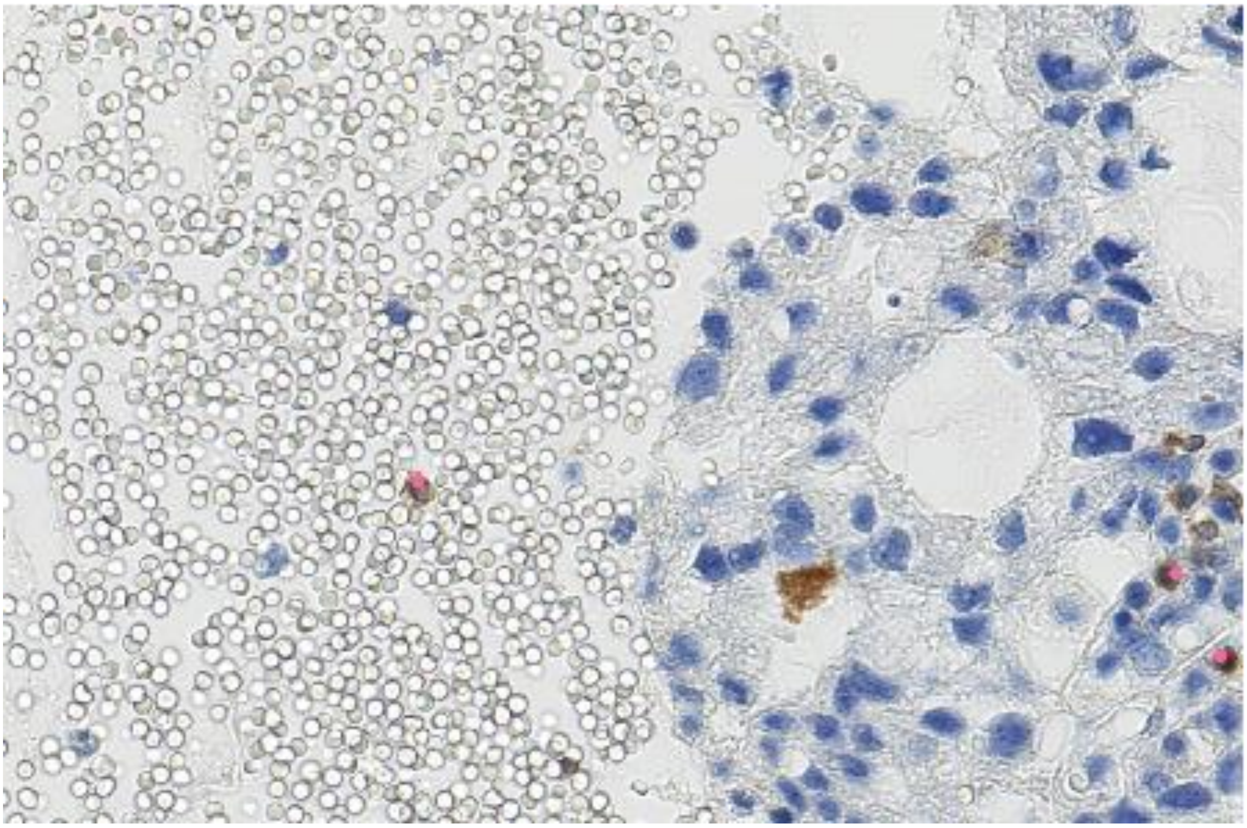

Supplement: Supplemental Information 1 — Figure S1 indicates patches which model could not discriminate properly, such as, false positive and false negative cases. Figure S2 shows an overview over variety of artifacts, which are encountered within the stained lung adenocarcinoma tissue slides. These images are extracted from the validation image set. In general, artifacts are not recognized as cells by our method. The red dots indicate detected cells. [file peerj-07-6335-s001.pdf]
